# Engineering Enzymatically Activated “Molecular Grenades” for Cancer

**DOI:** 10.18632/oncotarget.562

**Published:** 2012-07-21

**Authors:** Samuel R. Denmeade, John T. Isaacs

**Affiliations:** The Sidney Kimmel Comprehensive Cancer Center at Johns Hopkins, Baltimore MD; The Sidney Kimmel Comprehensive Cancer Center at Johns Hopkins, Baltimore MD

## Rationale for Cancer Drug Development

Cancer drugs are a unique class of therapeutic agents. Like all therapeutics, they interfere with pathophysiological processes. Unlike other therapeutics, however, interference with these processes in cancer patients is intended to produce death of malignant cells without killing or injuring the non-malignant cells in normal host tissues. This is a daunting engineering challenge. Remarkably, modest successes, and even cures, are achieved through the use of non-selective, proliferation dependent chemotherapeutic poisons in a limited number of cancer types due to their higher proliferative rate and lower apoptotic threshold compared to normal tissues [1]. The price for such non-selectivity, however, is dose-limiting host toxicities, particularly myelosuppression, which greatly limits therapeutic efficacy against the more common solid malignancies. These limitations have lead to a profound re-thinking about the rationale for cancer drug development.

Cancer cells often acquire an addiction to specific oncogenic signaling pathways [2]. Based upon this acquisition, a new class of targeted drugs has emerged designed to selectively inhibit only particular oncogenic signaling protein targets. In theory, such highly selective oncogene-based inhibitors target growth suppression and/or death of individual oncogene-addicted cancer cells sparing host normal cells. As a class, these new oncogene-targeted inhibitors are less toxic than chemotherapeutics, but they are not without side effects. More significantly, their therapeutic efficacy is limited by heterogeneity within the cancer cell population with regards to addiction to the specific oncogenic signaling resulting in drug resistance [3, 4].

## Why Use Enzymatically Activated Molecular Grenades?

A strategy to overcome such tumor cell heterogeneity based therapeutic resistance is to develop a drug that acts like a “molecular grenade” in that it is designed to “detonate” upon release of a non-selective toxin restrictively within the extracellular microenvironment of metastatic sites of cancer. The chemical engineering requirements for optimizing the non-selective toxin are that it should be: 1) highly cell penetrant (i.e., lipophilic), 2) a potent inhibitor of an essential intracellular process required for survival by all cell types, and 3) capable of peptide bond formation with a specifically engineered carrier peptide. The amino-acid sequence of the carrier peptide is optimized to allow for coupling to the non-selective toxin via a peptide bond to produce a water soluble non-cell penetrant drug which is efficiently hydrolyzed releasing the cell penetrant toxin only by a defined protease whose expression is restricted to metastatic sites of cancer. Thus, when the drug is infused, it distributes systemically throughout the body but can only be activated (i.e. detonated) within the extracellular fluid by a plasma membrane protease expressed by at least a subset of cells within cancer sites, but not by cells in normal tissue. Within tumor sites, the protease “pulls the pin” on the grenade by proteolytically releasing the cell penetrant toxin. Once liberated, the toxin rapidly penetrates cells in its immediate vicinity due to its lipophilicity and does not re-enter the circulation, thus restricting its non-selective toxicity to the cancer microenvironment. The advantage of such selective extracellular hydrolysis is that only a fraction of the cells need to express the enzyme since its continuous activity amplifies the level of cell penetrant toxin liberated into the extracellular fluid shared by all cells within the metastatic site. This amplification minimizes the problem of tumor cell heterogeneity by inducing a substantial “bystander effect” in which, like a detonated grenade, all cells within the tumor site including both malignant and infiltrating host supportive cells are killed, even those that do not express the activating enzyme. Thus, development of resistance is retarded without simultaneously producing non-selective host toxicity.

As examples of this molecular grenade strategy, we rationally engineered a drug platform based upon covalently coupling a chemically modified amino-acid containing thapsigargin (TG) analog toxin to a series of enzyme cleavable peptide carriers. Amino-acid containing TG analogs are intentionally selected because they are non-selective toxins that have low nM ability to kill all cell types by inducing Endoplasmic Reticulum (ER) stress through their inhibition of the Sarcoplasmic/Endoplasmic Reticulum Calcium ATPase (SERCA) pump, a critical intracellular protein whose normal function is required by all cell types to maintain viability [5]. Using this approach, we designed TG drugs that are restrictively hydrolyzed by the carboxypeptidase Prostate-Specific Membrane Antigen (PSMA) or the serine prolyl protease Fibroblast Activation Protein (FAP). PSMA is highly expressed by both normal and malignant prostate cancer cells. However, PSMA is also expressed on the surface of tumor endothelial cells within the majority of solid cancers [6]. In a recent study we demonstrated that G202, a PSMA-activated drug, selectively killed PSMA-producing cells in vitro and produced significant regression of a panel of human cancer xenografts. G202 is currently being tested in early phase clinical trials [6]. As a second approach, FAP was selected as a pan-tumor target based upon its universal expression by cancer-activated fibroblasts (CAFs) in contrast to the lack of its expression by fibroblasts within normal tissues [7]. FAP is also expressed by bone marrow derived mesenchymal stem cells that infiltrating metastatic sites of cancer [7]. We designed and synthesized FAP-activated thapsigargin drugs and documented that these also have profound antitumor efficacy against human cancer xenografts (8). This response is due to the selective killing of stromal elements within these xenografts involving a bystander effect against FAP-negative tumor endothelial cells and pericytes [8].

## Future Directions

Animal toxicology and clinical phase I trial data have documented that the PSMA-activated thapsigargin drug, G202 is non-myelosuppressive (6). This lack of myelosuppression facilitates G202 combination with a variety of additional clinically approved drugs. For example, thapsigargin's ability to induce ER stress raises its potential for synergy when combined with radiation and cytotoxic chemotherapies. Finally, G202-based delivery of the thapsigargin analog causes marked reduction of expression of the androgen receptor (AR) in prostate cancer cells and the estrogen receptor (ER) protein in breast cancer [5, 6]. These results suggest that combination therapy with anti-androgen/estrogens and G202 could be synergistic against prostate and/or breast cancer. These combinatorial approaches are currently under pre-clinical evaluation in our laboratories.
